# An evaluation study of direct economic losses from surgical site infections in adults: structural equation modeling

**DOI:** 10.3389/fpubh.2024.1514444

**Published:** 2025-01-13

**Authors:** Qiuxia Zuo, Di Liu, Baoji Dong, Yuan Zhou, Kexin Zhao, Ping Tian

**Affiliations:** ^1^School of Nursing, Xinjiang Medical University, Urumqi, Xinjiang, China; ^2^Infection Management Department, The Fifth Affiliated Hospital of Xinjiang Medical University, Urumqi, Xinjiang, China; ^3^Infection Management Department, The Sixth Affiliated Hospital of Xinjiang Medical University, Urumqi, Xinjiang, China; ^4^Infection Management Department, Xinjiang Uygur Autonomous Region People's Hospital, Urumqi, Xinjiang, China; ^5^Health Care Research Center for Xinjiang Regional Population, Urumqi, Xinjiang, China

**Keywords:** surgical site infections, direct economic loss, recursive system modeling, influencing factors, structural equation

## Abstract

**Introduction:**

Surgical site infection (SSI) represents a significant postoperative complication, resulting in extended hospital stays and substantial economic burdens. Previous research on the direct economic impact of SSIs using recursive systems modeling is limited. This study aims to quantify the direct economic losses attributable to SSIs and to dissect the various factors to these losses.

**Methods:**

A retrospective 1:1 matched case–control study was conducted from January 2023 to March 2024 in three tertiary hospitals in Xinjiang, China. Patients with SSIs were matched on a 1:1 basis by hospital, department, age (±5 years), sex, primary diagnosis, and procedure with controls to form case and control groups. Wilcoxon Signed Ranks Test was utilized to quantify the direct economic loss from SSIs. Influencing factors were analyzed using a recursive system model.

**Results:**

Among the 74,258 patients surveyed, 226 developed SSIs, resulting in an infection rate of 0.3%. The total direct economic loss from SSIs at three hospitals was $467,867, with an average loss of $1,364.37 per SSI patient. SSI patients experienced hospital stays 11 days longer than uninfected patients. Multivariate linear regression identified the duration of hospital stay, catheter and ventilator usage, age, number of surgeries, and duration of antibiotic treatment as influencing factors. Recursive system modeling revealed the indirect contributions of the number of surgeries (indirect effect: 0.074), antibiotic use for 17–36 days (indirect effect: 0.063) and ≥ 37 days (indirect effect: 0.045), and debridement procedures (indirect effect: 0.054), as well as the direct contributions of hospital days (direct effect: 0.276), indwelling catheter days (direct effect: 0.260), ventilator days (direct effect: 0.221), and age (direct effect: 0.182).

**Conclusion:**

Recursive system modeling helped identify the key factors influencing the economic losses from SSIs. These findings provide a theoretical basis for healthcare departments to develop targeted policies.

## 1 Introduction

Surgical site infections (SSIs) rank among the most frequently reported hospital-acquired infections (HAIs). The Center for Disease Control and Prevention (CDC) characterizes SSIs as postoperative infections occurring within 30 days of surgery, or within 1 year if permanent implants are involved. These infections include superficial incisional, deep incisional, and organ/space infections (1). It represents a significant complication for patients post-surgery and poses a major challenge to healthcare-associated infections globally. The three primary HAIs are respiratory infections, urinary tract infections, and SSIs, with the latter leading to readmissions, re-operations, extended antibiotic use, and prolonged hospital stays (2). These infections not only compromise patient health and cause mental stress to patients and their families but also lead to economic losses and resource wastage in the healthcare system.

Currently, the rising number of surgical procedures has elevated SSIs to a critical public health issue (3). The global incidence of SSI is 2.5% (4). Incidence rates are 0.9% in the United States, 3.6% in Australia, 2.9% in China, 2.8% in Africa, and 6.1% in low- and middle-income countries (2, 5–7). A European cohort study (8) revealed an SSI incidence of 5.0%. Direct economic costs of SSIs primarily consist of additional medical expenses and extended hospital stays required for treating these infections (9). A French cohort study reported that the average cost of treating an SSI is approximately €1,814, resulting in annual treatment costs ranging from $10,443 to $25,546 in France (10). In the United States, direct economic losses attributable to SSIs range from $93 million to $112 million annually (11). In Germany, the mortality rate for SSI patients is more than double that of non-infected patients, and the average hospital stay extends by 16 days for those affected by SSIs (12). Studies consistently demonstrate that SSIs are associated with increased costs.

Therefore, it is crucial to understand the hospitalization costs of surgical patients and the factors that influence them in order to manage and mitigate the direct economic losses from SSIs. Structural equation modeling has been increasingly utilized to analyze hospital-acquired infections, particularly hospitalization costs. Studies have demonstrated both direct and indirect relationships between hospitalization costs and influencing factors within a well-defined recursive system structure (13). Using linear regression analysis alone yields only the direct relationships between influencing factors and hospitalization costs, thereby overlooking the complexity and hierarchical nature of each indicator (14). However, the recursive system model in structural equations accounts for all variables in multiple dependent variable equations simultaneously, clearly delineating the complex hierarchical relationships among hospital indicators (15).

Current research lacks studies employing recursive system modeling to analyze the factors influencing the direct economic losses from SSIs. Thus, this paper seeks to identify the key factors for controlling the direct economic losses of SSIs by examining these losses after surgery; analyzing the factors influencing these losses in surgical patients using the recursive system model. This research aims to provide a scientific foundation for health-related departments to develop relevant policies.

## 2 Methods

### 2.1 Study design

A retrospective 1:1 matched case–control study without blinding was conducted to collect data on general information, relevant variables, and hospitalization costs of SSI patients from January 1, 2023, to March 31, 2024, at three tertiary hospitals in Xinjiang. Patients with SSI formed the case group, while those without SSIs formed the control group. Matching occurred within the same hospital and department, aligning cases and controls by age (±5 years), sex, primary diagnosis, and procedural consistency. The control group was randomly selected from patients meeting these criteria, and the direct economic losses from SSI was calculated. Risk factors for economic losses of SSI in the matched case group were also analyzed using recursive system modeling.

### 2.2 Participants

Participants were patients diagnosed with SSI between January 2023 and March 2024 at three tertiary hospitals in a city in Xinjiang. Inclusion criteria were: ① Diagnosis of SSI according to the 2024 CDC NHSN Patient Safety Manual (1); ② Age ≥ 18 years; ③ Complete clinical data. Exclusion criteria included: ① Presence of community-acquired infections at admission; ② Pregnancy. This retrospective study received approval from the Ethics Committee of the Fifth Affiliated Hospital of Xinjiang Medical University (approval number XYDWFYSk-2022-12). The committee confirmed that the study conformed to the ethical standards of the 2013 Declaration of Helsinki. Due to the observational nature of this study, informed consent was waived in all participating hospitals. All clinical data were anonymized and de-identified prior to analysis.

### 2.3 Data collection

Data were retrospectively collected through the hospital infection surveillance systems: HIS system and Apricot Grove system. Collected variables included gender, age, underlying diseases (such as hypertension, diabetes, coronary artery disease, pulmonary disease), number of hospital days, surgeries, days of antimicrobial drug use, pathogen cultures, days of surgical incision drainage, indwelling catheterization, respiratory use, and central venous placement. Hospitalization costs were segmented into categories including total cost, treatment, laboratory, examination, nursing, rehabilitation, general medical services, medicine, surgical blood transfusion, proprietary Chinese medicine and herbal medicine, consumables, and other costs.

### 2.4 Recursive system model

The recursive system model, a specific type of simultaneous equation model (16), incorporates both endogenous and exogenous variables. Endogenous variables are treated as dependent, while exogenous variables act as independent variables; exogenous variables influence the model independently, and endogenous variables can also serve as exogenous variables in other equations. Direct effects are exerted by a variable on the dependent variable, and indirect effects are mediated through other variables (16).

$$Y_{1}=\gamma_{11}\;X_{1}+\gamma_{12}\;X_{2}+\ldots \ldots +\gamma_{1k}\;X_{k}+E_{1}$$

$$Y_{2}=\gamma_{21}\;X_{1}+\gamma_{22}\;X_{2}+\ldots \ldots +\gamma_{2k}\;X_{k}+\beta_{21}\;Y_{1}+E_{2}$$

(1) $$\begin{array}{l} Y_{n}=\gamma_{n1}\;X_{1}+\gamma_{n2}\;X_{2}+\ldots \ldots +\gamma_{nk}\;X_{k}\\ +\beta_{n1}\;Y_{1}+\ldots \ldots +\beta_{n,n-1}\;Y_{n-1}+E_{n} \end{array}$$

Equation 1 outlines the recursive system model where Y is the endogenous variable, X is the exogenous variable, and E is the error term.

### 2.5 Statistical analysis

SPSS 26.0 software was utilized for statistical analysis. Count data were subjected to normality and chi-squared tests. Non-normally distributed data were presented as median (M) and interquartile range (Q). The differences in hospitalization costs and hospital days between the case and control groups were assessed using a two-independent-samples paired rank sum test (Wilcoxon Signed Ranks Test). Multiple linear regression analyzed the factors influencing the direct economic losses from SSIs. AMOS 26.0 software was used to construct a recursive system model. Non-normally distributed quantitative data required logarithmic transformation, to analyze the factors influencing the direct economic losses of SSI patients. The significance level was set at *α* < 0.05.

## 3 Results

### 3.1 Surgical site infection rate and incidence across departments

**Figure 1:**
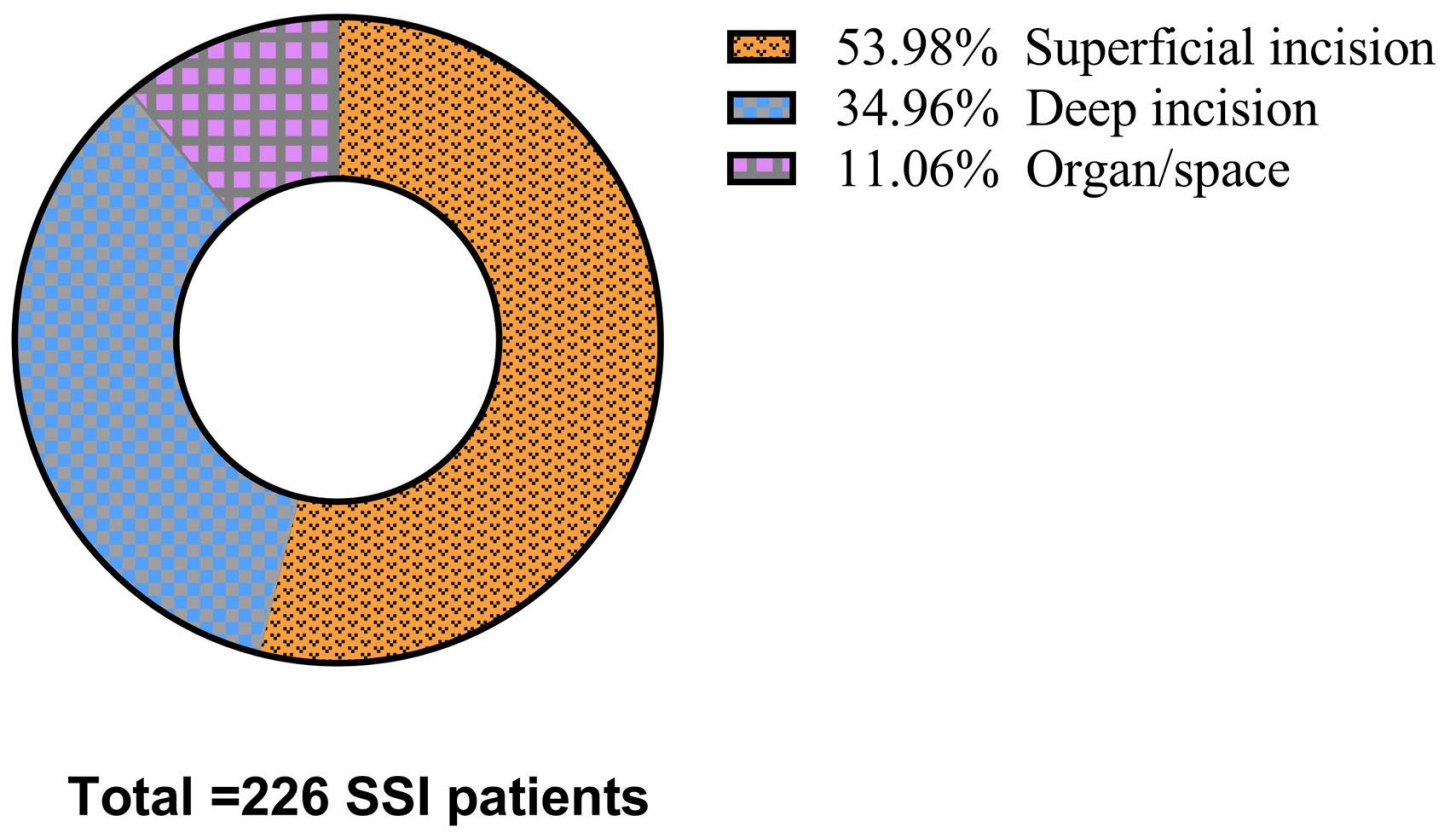
Distribution of different infection sited.

From January 2023 to March 2024, a total of 74,258 surgical procedures were performed across three hospitals, resulting in 226 SSIs, with an overall postoperative SSI rate of 0.3% (226/74,258). Of these, 21.79% (49/226) underwent debridement. Superficial incision SSIs accounted for 54% (122/226), deep incision SSIs for 35% (79/226), and organ/space SSIs for 11% (25/226), as detailed in Figure 1. Table 1 demonstrates the distribution of SSIs across various surgical departments: orthopedics recorded 86 SSIs out of 22,803 procedures (0.38% SSI rate); cardiac surgery had 9 SSIs from 998 procedures (0.90%); thoracic surgery reported 9 SSIs from 1,936 procedures (0.46%); gynecology had 9 SSIs out of 10,564 procedures (0.09%); neurosurgery had 8 SSIs from 1,339 procedures (0.60%); the breast and thyroid department had 8 SSIs out of 5,179 procedures (0.15%); minimally invasive, hernia, and abdominal wall surgeries reported 6 SSIs from 3,817 procedures (0.16%); gastrointestinal surgery saw 4 SSIs from 2,241 procedures (0.18%); anorectal surgery had 4 SSIs from 2,444 procedures (0.16%); vascular surgery had 4 SSIs from 2,251 procedures (0.18%); maxillofacial surgery reported 3 SSIs from 2,058 procedures (0.15%); hepatobiliary and pancreatic surgery reported 3 SSIs from 3,520 procedures (0.09%); urology reported 3 SSIs from 7,702 procedures (0.04%); emergency trauma surgery saw 2 SSIs from 1,232 procedures (0.16%); plastic and reconstructive surgery had 2 SSIs from 1,594 procedures (0.13%); the Burn Wound Repair Department reported 1 SSI from 1,813 procedures (0.06%); and ophthalmology had 1 SSI from 2,817 procedures (0.04%).

**Table 1:** Surgical site infections by department.

Department name	Number of surgeries	Number of SSI	SSI rate (%)
Orthopedics	22,803	86	0.38
Cardiac surgery	998	9	0.90
Thoracic surgery	1,936	9	0.46
Gynecology	10,564	9	0.09
Neurosurgery	1,339	8	0.60
Breast and thyroid surgery	5,179	8	0.15
Minimally invasive, hernia and abdominal wall surgery	3,817	6	0.16
Gastrointestinal surgery	2,241	4	0.18
Anorectal surgery	2,444	4	0.16
Vascular surgery	2,251	4	0.18
Maxillofacial surgery	2,058	3	0.15
Hepatobiliary and pancreatic surgery	3,520	3	0.09
Urology	7,702	3	0.04
Emergency trauma surgery	1,232	2	0.16
Plastic and reconstructive surgery	1,594	2	0.13
Burn wound repair	1,813	1	0.06
Ophthalmology	2,817	1	0.04

### 3.2 Direct economic loss of SSI patients

A total of 162 pairs were successfully matched, excluding six minors, five women in labor, one individual with incomplete clinical information, and 52 unmatched cases (no uninfected individuals met the matching criteria of the infected group). As indicated in Table 2, the average direct economic loss per SSI patient was $1,364.37. The rank sum test for all cost categories between the case and control groups revealed no significant differences in rehabilitation costs, costs of proprietary and herbal medicines, and consumable costs; however, significant differences were observed in total hospitalization costs, treatment costs, laboratory tests, examination costs, nursing care costs, general medical service costs, medicine costs, surgical transfusion costs, and other costs (*p* < 0.05). The median hospital stay was 26.5 days for the case group compared to 15 days for the control group, with this difference being statistically significant (Z = −8.489, *p* < 0.001). SSI patients experienced a hospital stay 11 days longer than those in the control group.

**Table 2:** Various economic losses for SSI (USD).

Sports event	Control subjects	Case group	Difference (the result of subtraction)	*Z*	*p*
	*M*	*M*	*M*		
Hospital costs	4,814.30	6,623.97	1,364.37	−3.555	<0.001
Treatment costs	471.87	717.33	183.40	−4.018	<0.001
Laboratory costs	385.28	694.26	215.95	−5.343	<0.001
Examination costs	476.70	567.49	101.43	−2.047	0.041
Nursing costs	44.59	90.37	43.96	−7.701	<0.001
Rehabilitation costs	0.00	0.00	0.00	−1.440	0.150
General medical services costs	98.70	189.56	77.98	−7.920	<0.001
Medication costs	312.83	593.23	159.20	−5.256	<0.001
Surgical blood transfusion costs	764.19	1,040.69	179.20	−3.593	<0.001
Cost of proprietary and herbal medicines	16.02	22.97	0.00	−1.613	0.107
Cost of consumables	1,622.88	1,659.79	104.59	−1.233	0.218
Other costs	0.00	0.00	0.00	−2.676	0.007
Days of hospitalization	15.00	26.50	11.00	−8.489	<0.001

SSI is surgical site infection, M is median.

### 3.3 Composition ratio of Total direct economic loss for patients with SSIs

**Figure 2:**
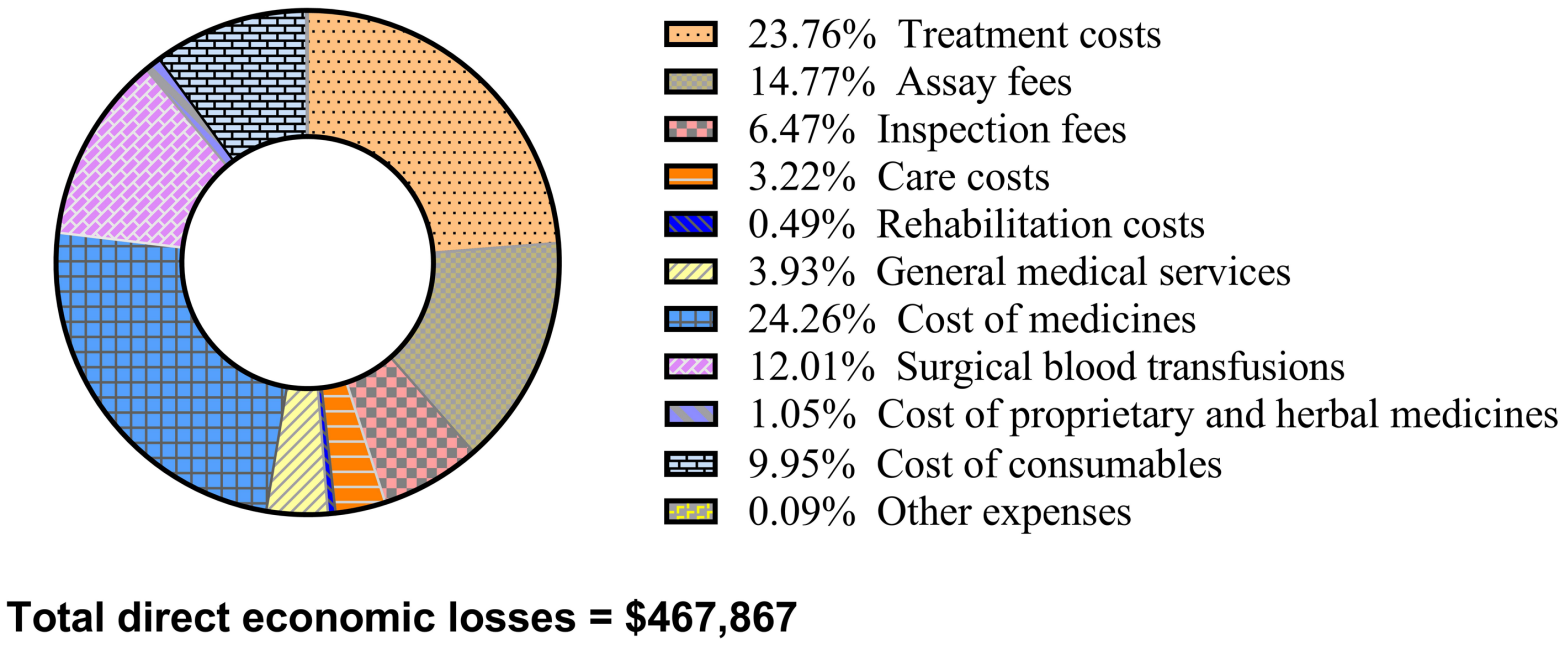
Component analysis of surgical site infection direct economic losses.

Figure 2 illustrates that the total direct economic loss for SSI patients in the three hospitals amounted to $467,867. The breakdown of costs is as follows: $113,487.63 (24.26%) for medications, $111,165.20 (23.76%) for treatments, $69,115.02 (14.77%) for laboratory tests, $56,200.34 (12.01%) for surgical blood transfusions, $46,561.16 (9.95%) for consumables, $30,281.32 (6.47%) for examinations, $18,370.13 (3.93%) for general medical services, $15,046.69 (3.22%) for nursing care, $4,933.31 (1.05%) for proprietary and herbal medicines, $2,282.56 (0.49%) for rehabilitation, and $401.80 (0.09%) for other expenses.

### 3.4 Exogenous variable screening results

Age, diabetes mellitus, hypertension, coronary artery disease, pulmonary disease, number of surgeries, debridement surgeries, culture of pathogenic bacteria, days of surgical incision drainage, days of mechanical ventilation, days of central venous catheterization, days of antibiotic use, and days of urethral catheterization (17) were selected as exogenous variables for the model. Categorical variables were treated as dummy variables and assigned values, as shown in Table 3.

**Table 3:** Assignments of the variables.

Variable code	Variant	Dummy variable	Assign a value to something
Y_1_	ln days of hospitalization		No
Y_2_	lnSSI direct economic losses		No
X_1_	Age		No
X_2_	Diabetes mellitus		0 = No 1 = Yes
X_3_	High blood pressure		0 = No 1 = Yes
X_4_	Coronary heart disease		0 = No 1 = Yes
X_5_	Lung Diseases		0 = No 1 = Yes
X_6_	Number of operations		Not have
X_7_	Debridement		0 = No 1 = Yes
X_8_	A culture of pathogenic bacteria		0 = No 1 = Yes
X_9_	Days of surgical incision drainage		Not have
X_10_	Days of mechanical ventilation		Not have
X_11_	Days of central venous catheterization		Not have
X_12_	Number of days on antibiotics	≤6 days	0 = ≤6 days
X_13_		7–16 days	0 = Other X_13_ = Yes
X_14_		17–36 days	0 = Other X_14_ = Yes
X_15_		≥37 days	0 = Other X_15_ = Yes
X_16_	Number of days on urinary catheter	≤5 days	0 = ≤5 days
X_17_		6–9 days	0 = Other X_17_ = Yes
X_18_		10–36 days	0 = Other X_18_ = Yes

The study identified that the number of hospitalization days serves as an intermediate variable, exerting both a direct and an indirect effect on hospitalization costs through other variables. Consequently, two linkage models were developed:

1. Model 1: Multiple Linear Regression Analysis with ln Hospitalization Days as the Endogenous Variable.

The distribution of information on the dependent variable was skewed and multiple linear stepwise regression analyses were performed after logarithmic transformation to screen for number of surgeries, use of antibiotics for 17–36 days, use of antibiotics for ≥37 days and debridement surgeries, with an adjusted R^2^ = 0.208, As shown in Table 4.

1. Model 2: Multiple Linear Regression Analysis with lnSSI Direct Economic Loss as the Endogenous Variable.

**Table 4:** Multiple linear regression analysis of factors affecting prolonged hospitalization days in patients with SSIs.

Model 1	Unstandardized coefficient		Standardized coefficient	t	*p*	95% CI	
	B	Std	β			Lower Limit	Limit
(Constant)	1.705	0.143		11.929	0.000	1.422	1.987
Number of surgeries	0.310	0.084	0.267	3.703	0.000	0.145	0.476
Debridement	0.476	0.179	0.194	2.656	0.009	0.122	0.830
Antibiotics 17–36 days	0.567	0.178	0.227	3.191	0.002	0.216	0.917
Antibiotics ≥37 days	1.300	0.562	0.163	2.314	0.022	0.190	2.409

Similarly, the distribution of the dependent variable information was skewed and multiple linear stepwise regression analyses were performed after logarithmic transformation to screen for the number of hospitalization days, age, number of days of mechanical ventilation, and use of urinary catheter for 10–36 days. Adjusted R^2^ = 0.259. As shown in Table 5.

**Table 5:** Multiple linear regression analysis of factors affecting direct economic losses from SSIs.

Model 2	Unstandardized coefficient		Standardized coefficient	t	*p*	95% CI	
	B	Std	β			Lower Limit	Limit
(Constant)	7.553	0.427		17.679	0.000	6.709	8.397
Hospital days	0.373	0.093	0.275	4.010	0.000	0.189	0.557
Age	0.017	0.006	0.181	2.660	0.009	0.004	0.030
Days on ventilator	0.086	0.027	0.220	3.178	0.002	0.033	0.140
Urinary catheter for 10–36 days	1.300	0.348	0.259	3.740	0.000	0.613	1.986

The recursive system model was fitted as follows:


$$\begin{array}{l} Y_{1}=\ln\;\left(\mathrm{days of differential hospitalization}\right)=\\ 1.705+0.310^{*}X_{6}+0.476^{*}X_{7}+0.567^{*}X_{14}+1.300^{*}X_{15}+E_{1}\text{.} \end{array}$$

$$\begin{array}{l} Y_{2}=\ln\;\left(SSI\;\mathrm{direct economic losses}\right)=\\ 7.553+0.373^{*}Y_{1}+0.017^{*}X_{1}+0.086^{*}X_{10}+1.300^{*}X_{18}+E_{2}\text{.} \end{array}$$

### 3.5 A pathway analysis of the direct economic losses due to surgical site infections and a calculation of the direct and indirect effects

**Figure 3:**
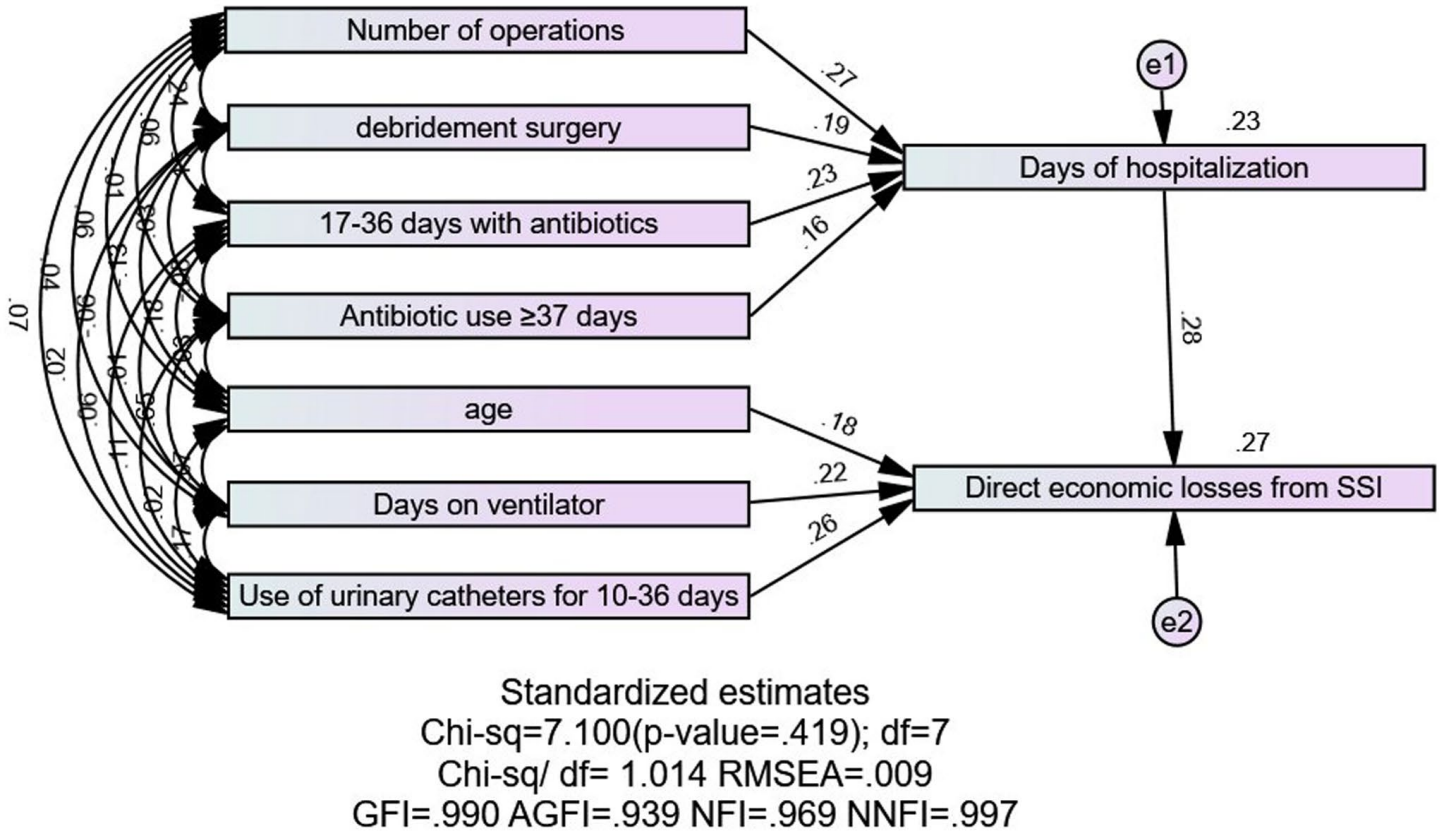
Path analysis of direct economic losses from surgical site infections.

Figure 3 illustrates a good model fit, indicated by Chi-sq = 7.100, df = 7, Chi-sq/df = 1.014, RMSEA = 0.009, GFI = 0.990, AGFI = 0.939, NFI = 0.969, and NNFI = 0.997. The number of surgeries, use of antibiotics for 17–36 days, debridement surgeries, and use of antibiotics for ≥37 days indirectly contributed to the number of hospital days for surgical SSI. Days of hospitalization, 10–36 days of urinary catheter use, days of ventilator use, and age directly contributed to the direct economic loss of patients with surgical SSI. As shown in Table 6, the total effect values of the factors, in descending order, were days of hospitalization, days of urinary catheter use, days of ventilator use, age, number of surgeries, use of antibiotics, and debridement surgeries.

**Table 6:** Contribution to the impact of direct economic losses of patients with SSIs by variable.

Exogenous variable	Dummy variable	Variable Code	Direct contribution	Indirect contribution	Total contribution	Ranking of total contributions
Hospital days		Y_1_	0.276	0	0.276	1
Number of surgeries		X_6_	0.000	0.074	0.074	5
Debridement		X_8_	0.000	0.054	0.054	7
Antibiotic use	Antibiotics 17–36 days	X_14_	0.000	0.063	0.063	6
	Antibiotics ≥37 days	X_15_	0.000	0.045	0.045	8
Age		X_1_	0.182	0	0.182	4
Days on ventilator		X_13_	0.221	0	0.221	3
Use of urinary catheters	Catheter 10–36 days	X_18_	0.260	0	0.260	2

## 4 Discussion

The occurrence of SSIs can lead to unplanned readmissions, debridement procedures, prolonged hospital stays, and delays in resuming normal work activities, incurring financial losses for patients (5). Despite interventions before and after surgery, the incidence of SSIs remains high, with an estimated 16,049 deaths annually (18). Critically, assessing and comparing the direct economic losses from SSIs with those from other diseases is essential for more focused attention and policy development. This study employed a case–control approach to analyze the direct economic losses from SSIs in surgical patients, assess both direct and indirect contributions of influencing factors using recursive system modeling, and calculate the overall public impact. Thus, understanding the factors influencing hospitalization costs for SSI patients is crucial for cost management and provides valuable insights for medical departments to implement strategies that reduce SSI rates and enhance patient safety.

The study revealed that the direct economic loss for SSI patients averaged $1,364.37. The median hospitalization cost was $6,623.97 for the case group versus $4,814.30 for the control group, significantly higher in the case group. The total direct economic loss for SSI patients in the three hospitals was $467,867, with medications accounting for the highest proportion at 24.26%. A systematic review involving 15 low- and middle-income countries and 16 European countries (19) found that economic losses from SSIs ranged from $174 to $29,610 in low- and middle-income countries, and from $21 to $34,000 in European countries. A French cohort study (10) reported the average cost of SSI treatment as approximately €1,814. Further, a study in China (20) noted that the direct economic loss from SSI in liver surgery patients was $5,408.20, with the highest proportion of costs attributed to medications, aligning with this study’s findings. The increased medical cost for SSI patients in a tertiary hospital in China was reported as $14,160.30 (21). Reasons for these variations include differences in study data, regional disparities in diagnostic and treatment capabilities, and the specific nature of the patient’s condition affecting treatment costs.

In the recursive system model analysis, the number of surgeries, debridement procedures, and antibiotic use were found to directly affect the number of hospital days and indirectly affect hospitalization costs. Studies have indicated (14, 22) that the number of surgeries directly impacts hospital stay lengths and indirectly influences hospitalization costs, aligning with this study’s findings. On one hand, multiple surgeries during hospitalization can lead to increased blood loss and surgical incisions, reducing immunity, slowing incision healing, and elevating infection risks, thereby extending the hospital stay. On the other hand, increased surgeries may incur additional fees for surgery, anesthesia, and postoperative care, raising hospitalization costs. Moreover, if a patient develops an SSI, it may necessitate readmission or debridement surgery, directly prolonging the hospital stay and indirectly escalating the patient’s financial losses.

Studies have demonstrated that using antibiotics for 17–36 days and ≥ 37 days can extend hospital stays. Prolonged antibiotic use may indicate medical staff concerns about potential SSIs, leading to added pathogen cultures and extended hospital stays (23). Slow detection of pathogen cultures may delay appropriate antibiotic administration, thus delaying infection symptom recovery and prolonging hospitalization. Furthermore, prolonged antibiotic use may promote drug-resistant bacteria development, reducing antibiotic effectiveness and necessitating the use of more advanced antibiotics, which wastes treatment time and extends hospital stays, indirectly impacting patients’ direct economic losses. Research shows (24) that extended postoperative antibiotic use does not decrease SSI rates. Hence, medical staff should enhance pathogenic delivery rates, improve drug sensitivity testing rates, and utilize antibiotics more judiciously to maximize therapeutic effects, shorten hospital stays, and minimize patients’ direct economic losses.

Rational prophylactic use of antibiotics is a crucial strategy to prevent SSIs. A meta-analysis (25) including 48 randomized controlled trials demonstrated that prophylactic antibiotics during surgery effectively reduce the incidence of SSIs. Flores-Yelamo et al. (26) found that a bundle package—consisting of prophylactic antibiotics, mechanical bowel preparation, perioperative glucose monitoring, adequate debridement, maintenance of intraoperative body temperature, use of a 2% chlorhexidine gluconate alcohol solution for skin disinfection, and changing instruments before wound closure—significantly lowered the risk of SSIs. Therefore, to prevent and control SSIs, healthcare professionals should assess the infection risk before surgery adequately. For high-risk patients, such as those with implants, long operating times, or who are immunocompromised, the use of appropriate prophylactic antibiotics can effectively reduce the risk of SSIs.

The study demonstrates that the number of hospitalization days serves as an intermediate variable, directly and indirectly affecting hospitalization costs. This correlation indicates that hospitalization costs increase with the length of stay. The duration of hospitalization was identified as a critical factor in the direct economic loss of SSI patients, corroborating findings from other studies (14, 22). Possible reasons include, on one hand, long-term hospitalized patients may present with more underlying diseases, complex conditions, compromised immunity, and extended physical rehabilitation needs, thereby prolonging their stay and increasing treatment and care costs. On the other hand, prolonged hospital stays heighten the risk of hospital-acquired infections, escalating antibiotic use and laboratory tests, such as cultures for pathogenic bacteria, thus directly increasing patients’ economic losses.

Research indicates that extended catheter retention correlates with increased hospitalization costs, aligning with other findings (27, 28). Postoperative urinary retention occurs in 2.5 to 43% of patients (29), typically resolved by temporary catheterization. Furthermore, pre-operative catheterization, often necessitated by anesthetic effects, may lead to prolonged postoperative urinary retention, thereby raising hospitalization costs. Prolonged catheter use may also cause urinary tract infections, increasing antibiotic usage and thus contributing to direct economic losses for patients. Additionally, for bedridden patients, catheters are sometimes requested by medical staff or family to manage daily living challenges, which extends catheter use and incurs further economic losses.

Studies also reveal that the number of days on mechanical ventilation directly impacts the direct economic loss of SSI patients, with longer ventilation periods correlating with higher healthcare costs (30, 31). This increase can be attributed to several factors: the aging population has elevated the clinical use of mechanical ventilation and its associated costs. Moreover, ventilated patients often have complex and critical conditions, leading to additional bedside exams, laboratory tests, and medications, which all raise hospitalization costs. Additionally, ventilated patients, often with reduced autonomy or in comatose states, require extensive life support and basic care, such as nasal feeding and skin care, further increasing care costs.

Studies have shown that the older the patient, the more significant the direct impact on the economics of SSI. However, other studies (13, 32) suggest that age indirectly affects hospitalization costs by increasing the length of stay, which does not entirely align with the findings of this study. On one hand, as age increases, patients often suffer from chronic conditions such as hypertension, diabetes, and cardiovascular diseases, necessitating additional pre-surgery tests and adjustments to manage blood sugar levels and meet surgical requirements, thereby directly increasing healthcare costs. On the other hand, declining bodily functions, slower metabolism, and decreased immunity in older patients may delay post-operative recovery and mobilization, leading to prolonged treatment and additional costs, thus directly affecting the patient’s economic losses.

### 4.1 Limitations

This study has several limitations. First, a retrospective design was used instead of a prospective approach, potentially underestimating the number of SSIs and limiting the ability to monitor correlations and statistical differences among certain variables. Second, the study focused on the direct economic losses from SSIs, excluding indirect losses. Additionally, these findings may not be applicable in other countries. Finally, the sample size was small. Future prospective studies with larger sample sizes are planned to further identify the key factors contributing to direct economic losses from SSIs.

## 5 Conclusion

The direct economic loss from SSI is substantial and influenced by various factors. The impact of each factor on the direct economic loss from SSI is ranked by contribution as follows: hospitalization days, indwelling urinary catheter days, ventilator days, age, number of surgeries, antibiotic use, and debridement surgeries. Therefore, hospitalization days are identified as the primary contributor to the direct economic loss from SSI. In clinical practice, efforts should be made to shorten hospital stays according to the patient’s condition, use urinary catheters judiciously and remove them as soon as feasible, and for surgical patients, medical staff should limit staged surgeries, use antibiotics judiciously, and focus on SSI prevention. By controlling modifiable factors, the incidence of SSI can be reduced, thus diminishing the direct economic losses to patients and providing a theoretical foundation for relevant departments to implement preventive and control measures against SSI.

## Data Availability

The data analyzed in this study is subject to the following licenses/restrictions: the data of this study are available from the corresponding author upon request. Requests to access these datasets should be directed to Qiuxia Zuo, zz1109431009@163.com.
